# Society of Surgical Oncology–American Society for Radiation Oncology–American Society of Clinical Oncology Consensus Guideline on Margins for Breast-Conserving Surgery With Whole-Breast Irradiation in Ductal Carcinoma In Situ

**DOI:** 10.1200/JCO.2016.68.3573

**Published:** 2016-08-15

**Authors:** Monica Morrow, Kimberly J. Van Zee, Lawrence J. Solin, Nehmat Houssami, Mariana Chavez-MacGregor, Jay R. Harris, Janet Horton, Shelley Hwang, Peggy L. Johnson, M. Luke Marinovich, Stuart J. Schnitt, Irene Wapnir, Meena S. Moran

**Affiliations:** Monica Morrow and Kimberly J. Van Zee, Breast Service, Memorial Sloan Kettering Cancer Center, New York, NY; Lawrence J. Solin, Albert Einstein Healthcare Network, Philadelphia, PA; Mariana Chavez-MacGregor, University of Texas MD Anderson Cancer Center, Houston, TX; Jay R. Harris and Stuart J. Schnitt, Harvard Medical School, Boston, MA; Janet Horton and Shelley Hwang, Duke University Medical Center, Durham, NC; Peggy L. Johnson, Advocate in Science, Susan G. Komen, Kansas City, KS; Irene Wapnir, Stanford University School of Medicine, Stanford, CA; Meena S. Moran, Yale School of Medicine, Yale University, New Haven, CT; and Nehmat Houssami and M. Luke Marinovich, Screening and Test Evaluation Program (STEP), Sydney School of Public Health, Sydney Medical School, The University of Sydney, New South Wales, Australia.

## Abstract

**Background:**

Controversy exists regarding the optimal negative margin width for ductal carcinoma in situ (DCIS) treated with breast-conserving surgery and whole-breast irradiation (WBRT).

**Methods:**

A multidisciplinary consensus panel used a meta-analysis of margin width and ipsilateral breast tumor recurrence (IBTR) from a systematic review of 20 studies including 7883 patients and other published literature as the evidence base for consensus.

**Results:**

Negative margins halve the risk of IBTR compared with positive margins defined as ink on DCIS. A 2 mm margin minimizes the risk of IBTR compared with smaller negative margins. More widely clear margins do not significantly decrease IBTR compared with 2 mm margins. Negative margins less than 2 mm alone are not an indication for mastectomy, and factors known to impact rates of IBTR should be considered in determining the need for re-excision.

**Conclusion:**

The use of a 2 mm margin as the standard for an adequate margin in DCIS treated with WBRT is associated with low rates of IBTR and has the potential to decrease re-excision rates, improve cosmetic outcome, and decrease health care costs. Clinical judgment should be used in determining the need for further surgery in patients with negative margins < 2 mm.

## INTRODUCTION

Breast-conserving therapy (BCT), defined as surgical excision of the primary tumor with a margin of surrounding normal tissue followed by whole-breast radiation therapy (WBRT), results in long-term cause-specific survival rates of greater than 95% for women with ductal carcinoma in situ (DCIS) as demonstrated in both randomized trials^1^ and observational studies.^2,3^ Although the addition of WBRT to surgical excision does not improve survival, it substantially reduces rates of ipsilateral breast tumor recurrence (IBTR), even among patients with small, non-high–grade DCIS.^1,4^ In the four early randomized trials of WBRT for DCIS, microscopically clear margins defined as no ink on tumor were required in three studies,^5-7^ but not in the fourth.^8^ These studies provide no information on whether more widely clear margins than no ink on tumor reduce rates of IBTR in patients having BCT.

Retrospective single-institution studies have suggested that a negative margin width of 1 cm or more may eliminate the reduction in IBTR seen with WBRT,^9^ leading some to conclude that larger margins are also beneficial in patients receiving WBRT. Despite the widespread use of BCT for DCIS, there is still no consensus on what constitutes an optimal negative margin width.^10^ As a consequence, approximately one in three women attempting BCT for DCIS undergo a re-excision.^11^ Re-excisions have the potential for added discomfort, surgical complications, compromise in cosmetic outcome, additional stress for patients and families, and increased health care costs, and have been associated with conversion to bilateral mastectomy.^12^

Since BCT was established, the landscape of DCIS management has evolved with advances in imaging and pathologic evaluation, and the availability of adjuvant endocrine therapy, resulting in a decline in IBTR rates.^13^ In view of these changes and the lack of consensus on what represents adequate negative margins in DCIS, the Society of Surgical Oncology (SSO), American Society for Radiation Oncology (ASTRO), and the American Society of Clinical Oncology (ASCO) convened a multidisciplinary margins panel (MP) to evaluate IBTR in relation to margin width. The primary question addressed was “what margin width minimizes the risk of IBTR in patients with DCIS receiving breast-conserving surgery?” The guideline developed from this consensus panel is intended to assist treating physicians and patients in the clinical decision-making process based on the best available evidence. The key findings of the guideline are summarized in Table 1.

**Table 1.**
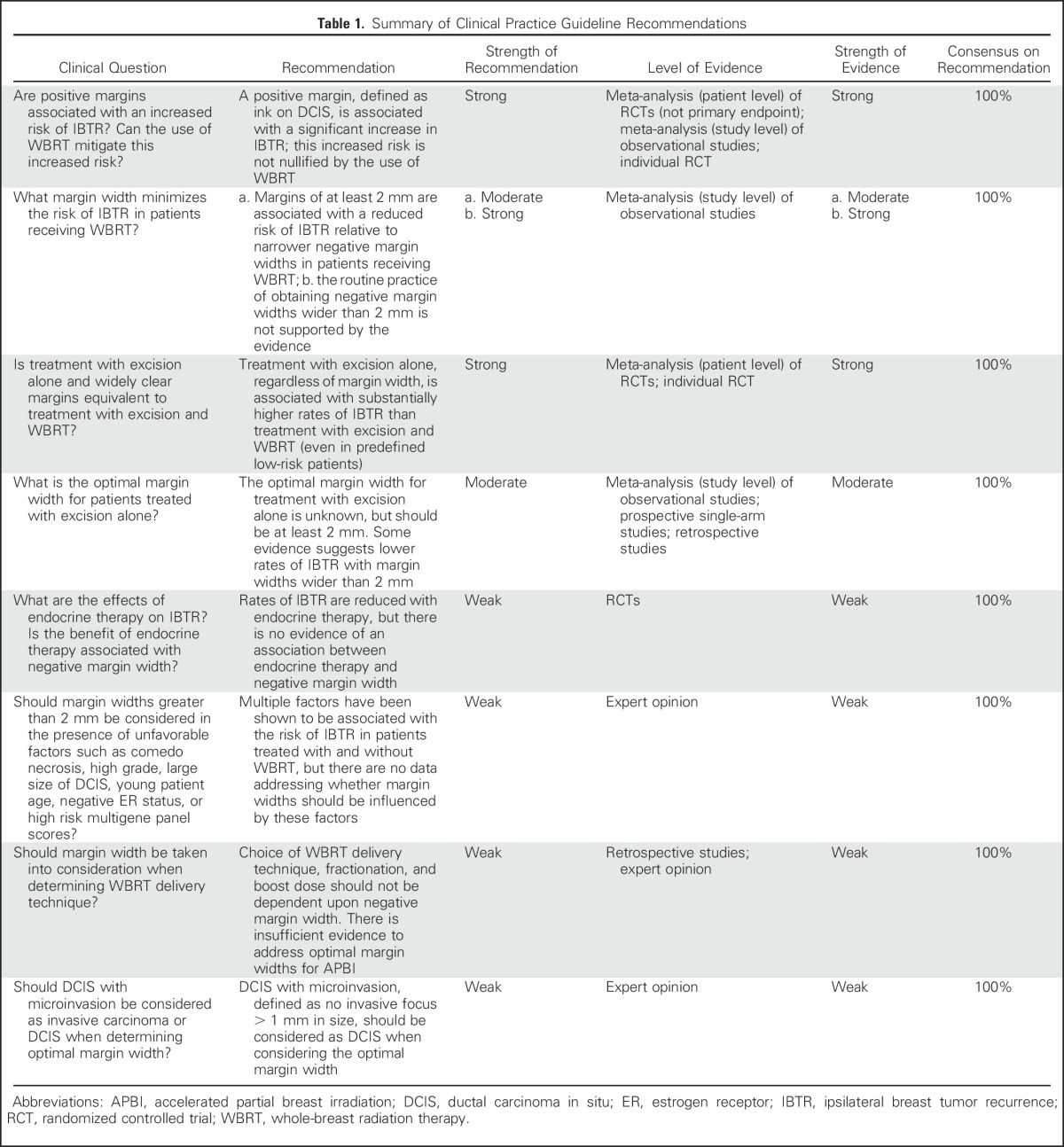
Summary of Clinical Practice Guideline Recommendations

## METHODS

The guideline development process was funded by a Susan G. Komen grant. Committee members were chosen by their respective organizations based upon interest and expertise in DCIS management (Table 2). Processes recommended in the Institute of Medicine report “Clinical Practice Guidelines We Can Trust”^14^ which were followed as part of the guideline development process included: (1) the development of a systematic review/study-level meta-analysis based on questions to be addressed by the MP to serve as the primary evidence base, with additional topic-specific literature reviews conducted by participants for questions not addressed in the meta-analysis; (2) the provision for each recommendation of a rating of the strength of the evidence and the strength of the recommendation; (3) the ascertainment of the level of agreement of panel members with each recommendation by vote, and the revision of recommendations to achieve greater than 90% consensus; and (4) the declaration by MP candidates of potential conflicts of interest before convening, and the obtaining of written disclosures at the consensus meeting. (The co-chairs deemed no MP members had conflicts that could influence the process/development of specific recommendations.)

**Table 2.**
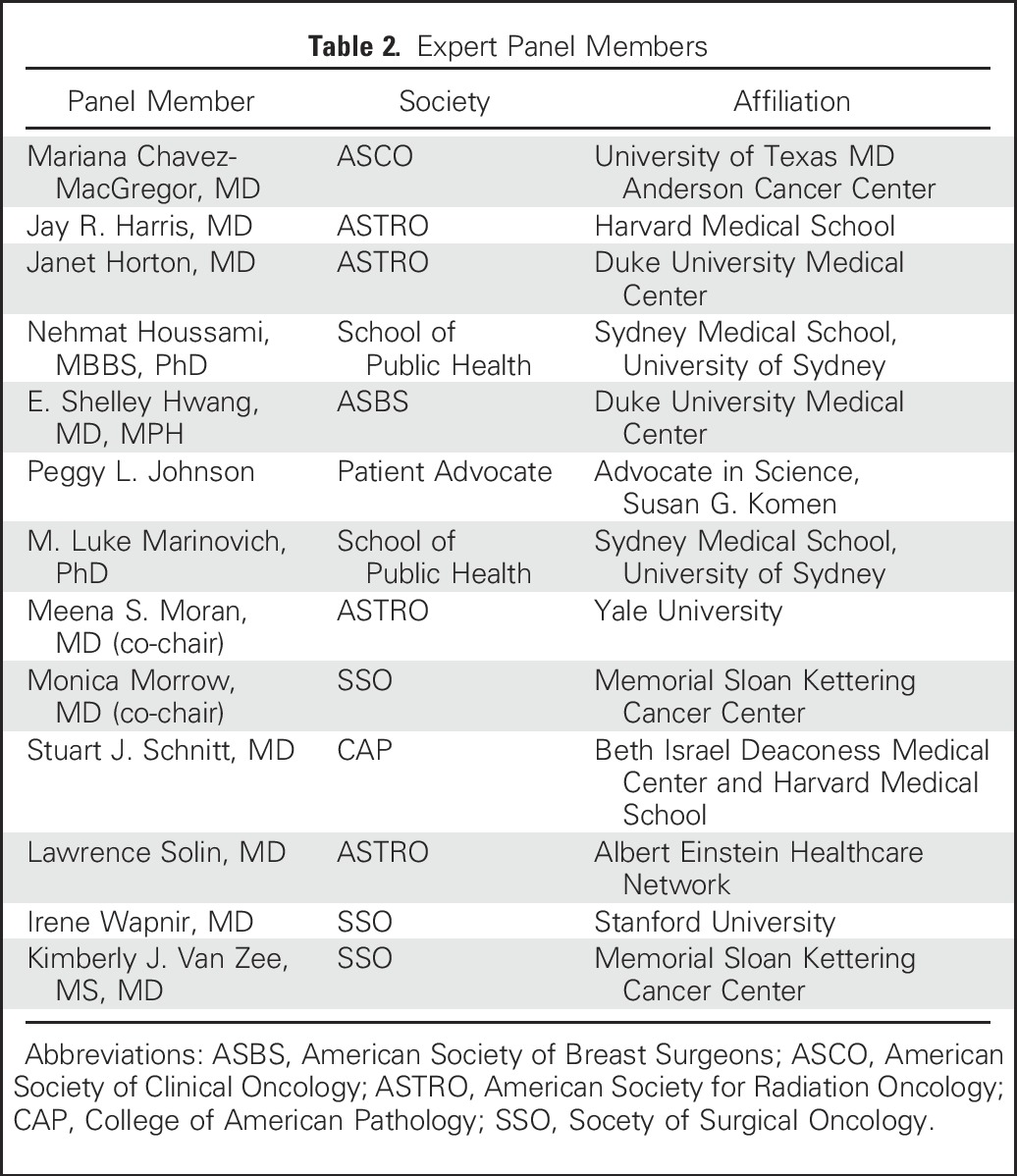
Expert Panel Members

The MP convened in November 2015; the resulting manuscript was approved by all panel members and externally reviewed, and feedback was incorporated. The final manuscript was approved by the SSO Executive Council, the ASTRO Board of Directors, and the ASCO Board of Directors, and endorsed by the Board of Directors of the American Society of Breast Surgeons. Patient-related materials will be available on the Susan G. Komen website (komen.org).

### Meta-Analysis

The methodology for the systematic review/meta-analysis has been published elsewhere.^15^ Briefly, using Preferred Reporting Items for Systematic Reviews and Meta-Analyses (PRISMA) and Institute of Medicine guidelines, EMBASE, MEDLINE, PREMEDLINE, and evidence-based medicine databases were searched in October 2014 for eligible studies. A summary providing details of the methodology and statistical approaches is provided in the Appendix. Analysis was performed using two different statistical approaches. In the frequentist approach, multiple margin cut points within studies, if reported, were condensed into a single cut point, while the Bayesian approach allowed for the use of multiple cut points.^16^ All reported odds ratio (ORs) were adjusted for study-specific median follow up time (to account for the inherent increased risk of IBTR with longer follow up) and are reported relative to positive (or positive/close) margins, or to a minimal negative margin (no ink on tumor or margin > 1 mm).^15^

### Inclusion/Exclusion Criteria

Studies that included a minimum of 50 patients with DCIS treated with local excision and reported IBTR in relation to microscopic margin widths with a minimum median follow up of 4 years were eligible.^15^

### Study Quality/Literature Limitations

All publications in the meta-analysis (except for two) were retrospective and provided observational data at the study level. The characteristics of these studies have been reported elsewhere.^15^

## RESULTS

The meta-analysis included 20 studies, 7883 DCIS patients with known margin status, and 865 IBTRs.^15^ The median proportion of patients receiving WBRT was 100% (interquartile range [IQR] 53.3%-100.0%), and the median proportion receiving endocrine therapy was 20.8% (IQR 0.0%-31.4%). The median follow up was 78.3 months, and the median incidence of IBTR was 8.3% (IQR 5.0%-11.9%). Due to heterogeneity in classification and reporting of margins data, both a frequentist analysis and a Bayesian network meta-analysis were conducted with sensitivity analyses. Characteristics of patients included in the studies are summarized in Table 3.^15^

**Table 3.**
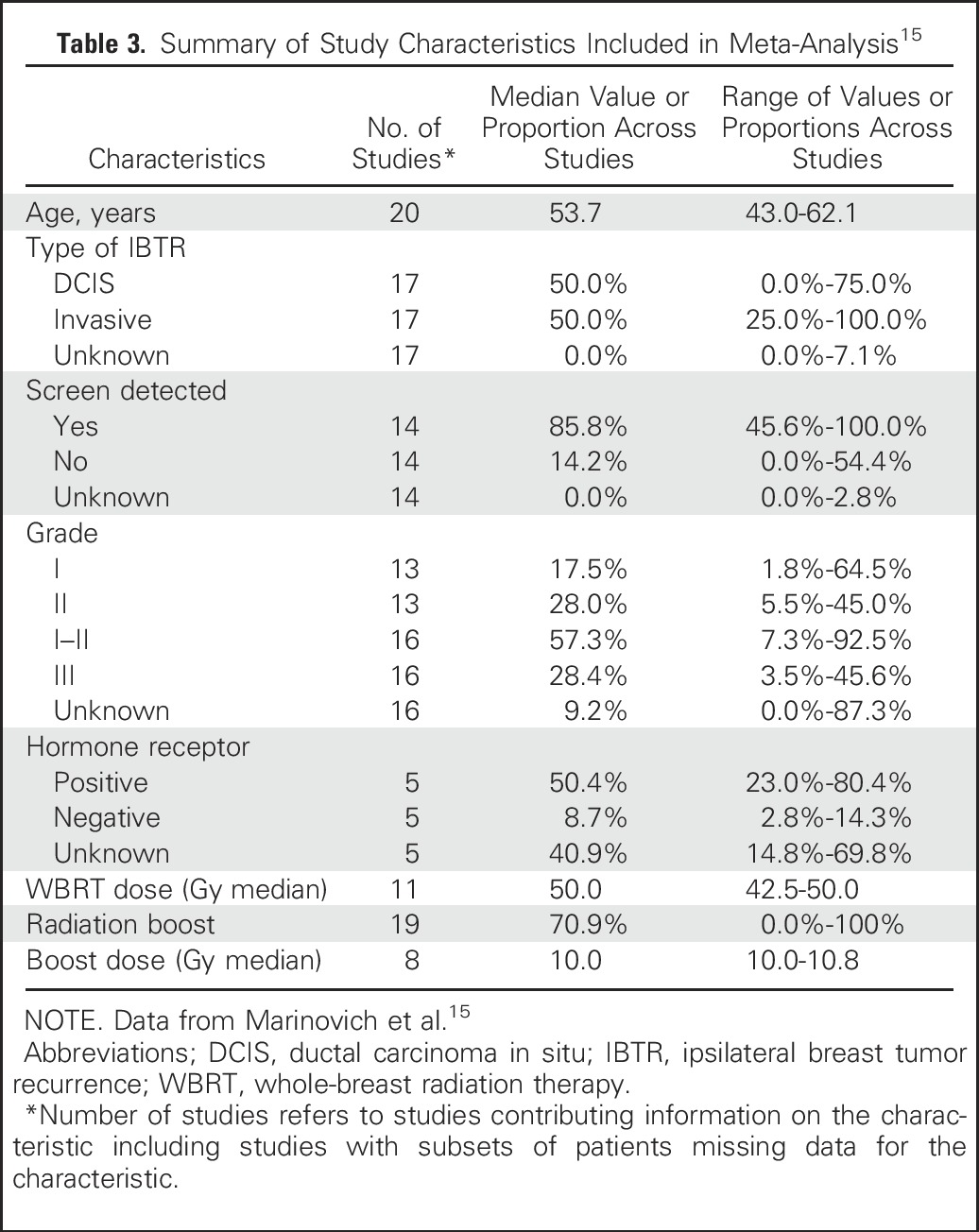
Summary of Study Characteristics Included in Meta-Analysis^15^

## GUIDELINE RECOMMENDATIONS

### Positive Margins

A positive margin, defined as ink on DCIS, is associated with a significant increase in IBTR. This increased risk is not nullified by the use of WBRT.

There is no debate that a positive margin, defined as the presence of ink from the specimen surface on ducts containing DCIS, implies a potentially incomplete resection and is associated with a higher rate of IBTR. In the Early Breast Cancer Trialists’ Collaborative Group (EBCTCG) meta-analysis of randomized DCIS trials,^1^ patients with positive margins had a twofold higher IBTR risk compared with patients with negative margins despite receiving WBRT (10-year IBTR rate 24% vs 12%), and approximately 50% were invasive recurrences. The relationship between margin status and WBRT was examined in a subset analysis of the National Surgical Adjuvant Breast and Bowel Project (NSABP) B-17 trial by central pathology review of 573 of 818 participants.^17^ The annual hazard rate for IBTR after lumpectomy alone was 8.1% for those with positive margins compared with 3.3% for patients with negative margins, reduced by WBRT to 2.7% and 1.2%, respectively. Positive margins were significantly associated with IBTR in a multivariate analysis of the long-term results of the European Organization for Research and Treatment of Cancer (EORTC) 10853 trial.^18^ In the meta-analysis of Marinovich et al using the Bayesian analytic approach, similar results were obtained.^15^ After adjustment for study-level follow up, patients with negative margins were significantly less likely to experience IBTR than patients with positive margins (OR 0.45, 95% credible interval [CrI] 0.30-0.62). Similar findings were observed in the frequentist analysis which necessitated combining positive and close margins (OR 0.53, 95% CI 0.45-0.62; *P* < .001). This result persisted after study-level adjustment for age, median recruitment year, grade of DCIS, use of WBRT, and use of endocrine therapy.

### Negative Margin Widths

Margins of at least 2 mm are associated with a reduced risk of IBTR relative to narrower negative margin widths in patients receiving WBRT. The routine practice of obtaining negative margin widths wider than 2 mm is not supported by the evidence.

To address the question of optimal negative margin width, the MP considered data on the distribution of DCIS in the breast. Studies of mastectomy specimens using whole organ sectioning and radiologic-pathologic correlation have demonstrated that while most cases of DCIS are unicentric, the involvement of the segment may be multifocal, with “gaps” of uninvolved tissue between foci of DCIS.^19^ Given this, a “negative margin” does not guarantee the absence of residual DCIS in the breast.

There are also technical limitations to margin assessment which impact the relationship between margin width and IBTR. For example, margins are artifactually narrower ex-vivo when specimens become flattened from lack of surrounding supportive tissue, a phenomenon exaggerated by compression for specimen radiography. Additionally, surface ink can track into deeper portions of the specimen, posing significant challenges in determining true margin location. Finally, tumor-to-ink distance on any single slide may not be representative of the entire specimen; an “adequate” margin on one section may become positive if additional or deeper sections are examined. Two common methods for margin evaluation include sectioning perpendicular to ink (to determine tumor-to-ink width) or en-face examination of shaved margins (where any residual tumor in the shaved specimen is considered a positive margin). While an advantage of the shaved method is greater surface-area examination, a known disadvantage is the higher frequency of margins categorized as positive that are, in comparison, negative by the perpendicular method, which may in turn result in unnecessary re-excision or even mastectomy.^20^ Specimen sampling is also highly variable, and even total sequential embedding results in only a small proportion (< 1%) of the specimen margins being examined.^21^ Together, these studies highlight the substantial variability in margin assessment irrespective of the technique used.

Despite variability in margin assessment, great emphasis has been placed on achieving specific negative margin widths. In the Marinovich frequentist meta-analysis, comparison of specific margin width thresholds (2 mm, 3 or 5 mm, and 10 mm) relative to negative margins defined as > 0 mm or 1 mm included 7883 patients with a median follow up of 6.5 years. The ORs for 2 mm (0.51 [95% CI 0.31-0.85], *P* = .01), 3 or 5 mm (0.42 [95% CI 0.18-0.97], *P* = .04), and 10 mm (0.60 [0.33-1.08], *P* = .09) showed comparable reductions in the odds of IBTR compared with > 0 mm or 1 mm, and pairwise comparisons found no significant differences in the odds of IBTR between the 2 mm, 3 or 5 mm, and 10 mm margin thresholds (all *P* > 0.40). In this model, the predicted 10-year IBTR probability for 2 mm negative margins was 10.1% (95% CI 6.3%-16.0%) compared with 8.5% for 3 or 5 mm (95% CI 3.6%-18.9%) and 11.7% (95% CI 6.7%-19.4%) for 10 mm margins. In the Bayesian network meta-analysis (Table 4),^15^ the ORs of incrementally wider negative margins relative to the positive margin category were 0.45 (95% CrI 0.32-0.61) for > 0 or 1 mm, 0.32 (95% CrI 0.21-0.48) for 2 mm, 0.30 (95% CrI 0.12-0.76) for 3 mm, and 0.32 (95% CrI 0.19-0.49) for 10 mm. Adjustments for clinically relevant covariates, including sensitivity analysis limited to studies using radiation therapy (RT), did not alter these mean OR estimates (Table 4). In this analysis, the relative odds ratio (ROR) of IBTR between the 10 mm and 2 mm threshold groups compared with positive margins was 0.99 (95% CrI 0.61-1.64), indicating no statistically meaningful difference.

**Table 4.**
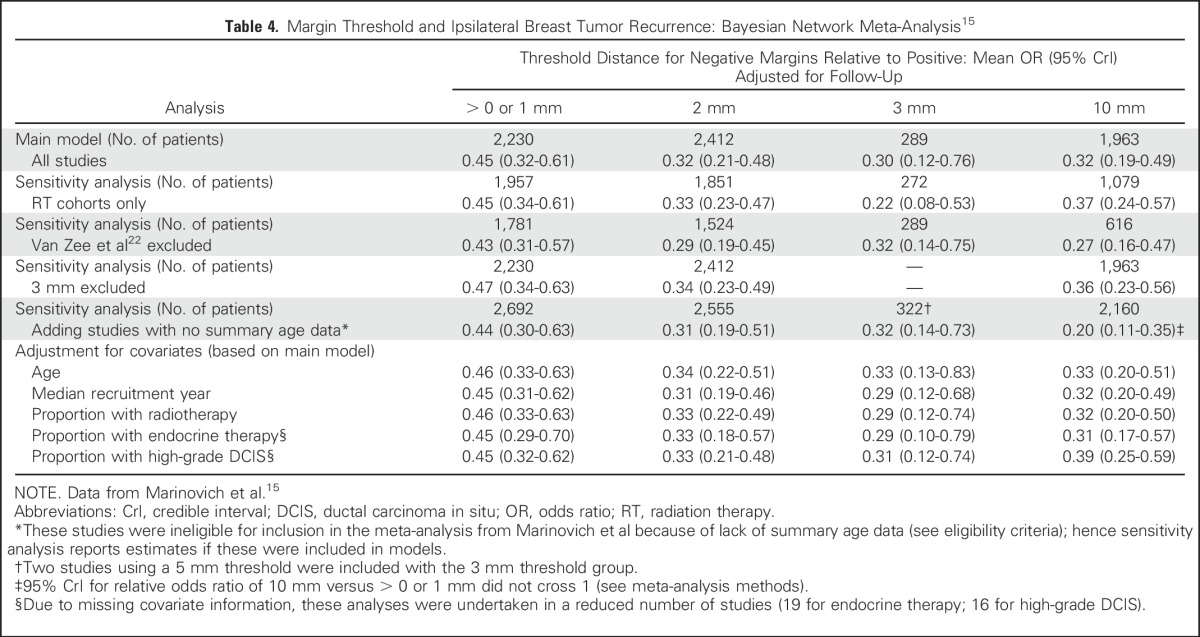
Margin Threshold and Ipsilateral Breast Tumor Recurrence: Bayesian Network Meta-Analysis^15^

The choice of the 2 mm threshold rather than > 0 (no ink on tumor) or 1 mm was based upon evidence of a statistically significant decrease in IBTR for 2 mm compared with 0 or 1 mm in the frequentist analysis (OR 0.51, 95% CI 0.31-0.85; *P* = .01) coupled with weak evidence in the Bayesian model of a reduction in IBTR with the 2 mm distance compared with smaller distances (ROR 0.72, 95% CrI 0.47-1.08). However, while the MP felt that there was evidence that the 2 mm margin optimized local control, clinical judgment must be used in determining whether patients with smaller negative margin widths (> 0 or 1 mm) require re-excision. Factors felt to be important to consider include assessment of IBTR risk (residual calcifications on postexcision mammography, extent of DCIS in proximity to margin, which margin is close [i.e., anterior excised to skin or posterior excised to pectoral fascia *v* margins associated with residual breast tissue]), cosmetic impact of re-excision, and overall life expectancy. The conclusion that re-excision could be selectively used with margins smaller than 2 mm was influenced by the high long-term rates of local control reported in the NSABP DCIS trials which required a margin of no ink on tumor^7^ as well as the study of Van Zee et al which, after adjusting for multiple covariates, found no difference in risk between margins of ≤ 2 mm and more widely clear margins in patients receiving WBRT.^22^

### Treatment With Excision Alone

Treatment with excision alone, regardless of margin width, is associated with substantially higher rates of IBTR than treatment with excision and WBRT, even in predefined low-risk patients. The optimal margin width for treatment with excision alone is unknown, but should be at least 2 mm. Some evidence suggests lower rates of IBTR with margin widths wider than 2 mm.

The EBCTCG DCIS meta-analysis showed that the 10-year IBTR rate for patients treated with excision alone was higher than with excision and WBRT, both for those with negative margins (26.0% vs 12.0%, *P* < .00001) and positive margins (48.3% vs 24.2%; *P* = .00004).^1^ The same proportional benefit for WBRT was seen in women treated with local excision and those having large sector resections. In the Radiation Therapy Oncology Group (RTOG) 9804 trial where patients with small, mammographically detected low-to-intermediate grade DCIS and margins ≥ 3 mm were randomized to excision alone or excision plus WBRT, 7-year IBTR rates were 6.7% and 0.9% (*P* = .0003), respectively.^4^ The prospective, multi-institutional Eastern Cooperative Oncology Group (ECOG) E5194 study of patients with low-risk DCIS treated with excision alone (negative margin width ≥ 3 mm) reported 12-year rates of IBTR of 14.4% for nonhigh grade DCIS ≤ 2.5 cm in size and 24.6% for high-grade DCIS ≤ 1 cm in size. However, IBTR rates did not differ significantly for margins < 5 mm, 5-9 mm, or ≥ 10 mm (*P* = .85).^23^ A prospective single-arm study of patients with mammographically detected DCIS ≤ 2.5 cm in size reported a 10-year IBTR rate of 15.6%^24^ despite requiring margins of ≥ 1 cm.^4^ In contrast, Van Zee et al found in 1266 patients treated with excision alone that 10-year IBTR rates were 16% for margins > 10 mm, and increased to 23% for margins between 2.1 and 10 mm, 27% for > 0-2 mm, and 41% for positive margins. After adjustment for multiple factors, margin width was a more highly significant predictor of IBTR (*P* < .0001).^22^ The MP felt that, overall, the heterogeneity of the evidence between the above-reported studies did not allow for a definitive recommendation for margin widths greater than 2 mm in patients foregoing RT.

### Endocrine Therapy

Rates of IBTR are reduced with endocrine therapy, but there is no evidence of an association between endocrine therapy and negative margin width.

Tamoxifen reduces the incidence of both IBTR and contralateral breast cancer, but the absolute benefit is relatively small.^7,25^ In the NSABP B-24 trial, patients treated with lumpectomy and WBRT were randomized to tamoxifen or placebo; 25% of the population had positive or unknown margins. The 15-year IBTR rate for the placebo group was 17.4% in those with positive margins compared with 7.4% for clear margins. Adjuvant tamoxifen lowered IBTR rates among those with positive margins to levels similar to those in the negative margin cohort (17.4% placebo, 11.5% tamoxifen); conversely, there was little impact of tamoxifen in the negative margin cohort (IBTR 7.4% placebo, 7.5% tamoxifen).^7^ Hence, the MP felt that while tamoxifen decreases IBTR in patients with positive margins, there was no evidence to suggest an association between negative margin width and the benefit of endocrine therapy.

### Patient and Tumor Features

Multiple factors have been shown to be associated with the risk of IBTR in patients treated with and without WBRT, but there are no data addressing whether margin widths should be influenced by these factors.

Young patient age has consistently been associated with IBTR, and tumor factors such as histologic pattern, comedo necrosis, and nuclear grade and size of DCIS also modify the risk of IBTR.^17,26,27^ More recently, unfavorable gene profile scores have also been associated with IBTR.^28,29^ However, there are no data addressing whether margin widths should be influenced by these factors, and this represents an appropriate area for further study.

### Radiation Delivery

Choice of WBRT delivery technique, fractionation, and boost dose should not be dependent upon negative margin width. There is insufficient evidence to address optimal margin widths for accelerated partial breast irradiation (APBI).

The vast majority of patients treated in the five prospective randomized DCIS trials of excision with or without WBRT received conventionally fractionated WBRT without a boost. Only one of the trials allowed the option of hypofractionated whole-breast RT (HWBRT) in addition to standard WBRT,^4^ and ≤ 10% of the patients in three of the trials received a boost.^6-8^ None of the randomized trials varied RT technique according to margin status, and intensity-modulated RT (IMRT) and accelerated partial breast irradiation (APBI) were not used.

There is no direct evidence from randomized trials to support the use of a boost to the primary tumor site for patients with DCIS, although in patients with invasive breast carcinoma, the long-term value of a boost in reducing IBTR has been demonstrated.^30^

Two ASTRO consensus guidelines have addressed technical issues in the setting of BCT. While largely focusing on invasive breast carcinoma, the ASTRO statement on HWBRT concluded there was insufficient evidence to recommend for or against HWBRT in the setting of DCIS.^31^ In the ASTRO statement on APBI, DCIS was placed into the “cautionary” group based on the lack of evidence from randomized trials, while noting that DCIS patients have been included in some retrospective cohort studies.^32^

Therefore, there is no evidence that margin width, in isolation, should determine radiation delivery technique, fractionation of WBRT, or use/dose of a boost. The MP considered the evidence base insufficient to address optimal margin width in APBI.

### DCIS in the Presence of Invasive Breast Cancer

DCIS with microinvasion, defined as no invasive focus > 1 mm in size, should be considered as DCIS when considering the optimal margin width.

There are two diagnoses for which there is overlap between the DCIS Margin Guideline and the Invasive Cancer Margin Guideline^33^: DCIS with microinvasion (DCIS-M) and invasive carcinoma associated with DCIS (extensive intraductal component [EIC] or lesser amounts of scattered DCIS). In DCIS-M, defined by the American Joint Committee on Cancer (AJCC) as the extension of cancer cells beyond the basement membrane with no focus more than 0.1 cm in greatest dimension,^34^ small retrospective studies suggest that rates of IBTR are similar to those seen with pure DCIS.^35,36^ In the absence of specific data to address margin width in DCIS-M, the MP, based on expert opinion, felt that DCIS-M should be considered as DCIS when considering the optimal margin width, given that the majority of the lesion is comprised of DCIS and that systemic therapy utilization for DCIS-M more closely reflects the treatment pattern for DCIS than for invasive carcinoma.

In contrast, when considering margin width for an invasive cancer with a DCIS component, regardless of extent, the MP felt that the invasive cancer guideline^33^ was applicable, primarily because the natural history and treatment of these lesions is more similar to invasive cancer than DCIS, even when the close margin contains DCIS. In particular, the vast majority of patients with invasive cancer receive systemic therapy, which remains less common for pure DCIS. The invasive cancer guideline^33^ did note that an EIC is a marker for a potential heavy burden of residual DCIS and that postexcision mammography, the presence of multiple close margins, and young patient age can be used to select patients who will benefit from re-excision. These statements echo the discussion of the MP regarding the use of re-excision in pure DCIS with margins < 2 mm discussed previously, and thus we believe that the guidelines are compatible.

## DISCUSSION

There are limitations to this guideline. It applies to patients with DCIS and DCIS-M treated with WBRT. The findings should not be extrapolated to DCIS patients treated with APBI or those with invasive carcinoma for whom a separate guideline has been developed.^33^ While studies including patients treated with and without WBRT were included in the meta-analysis, a meta-analysis of studies of treatment with excision alone was not conducted. Additionally, all of the studies included in the meta-analysis were retrospective. However, in the absence of any planned prospective randomized trials addressing the question of margin width and local recurrence, these studies represent the best available evidence for clinical decision making.

## Appendix

### Summary of Methodology for the Margins Meta-Analysis

The methods for the meta-analysis are described in full in Marinovich et al,^15^ and are summarized briefly below.

### Study Eligibility Criteria

Eligible studies enrolled ≥ 50 women with ductal carcinoma in situ (DCIS) undergoing breast conserving surgery (BCS); allowed calculation of the crude local recurrence (LR) rate by microscopic margin status; defined negative margins by a numeric threshold; reported mean or median age; and presented mean or median follow up of ≥ 48 months.

### Literature Search and Data Extraction

MEDLINE, PREMEDLINE, EMBASE, and ALL EBM REVIEWS were searched in October 2014. One investigator screened citations, with a sample independently screened by a second. Two investigators independently extracted data; disagreements were arbitrated by a third investigator.

### Statistical Analysis

#### Frequentist models (random effects logistic meta-regression).

Margins were dichotomised into positive/close versus negative margin status using one distance threshold per study (> 0 or 1 mm; 2 mm; 3 or 5 mm; 10 mm). The association between LR and margin status and distance was modeled using random effects logistic meta-regression. Odds ratios (ORs) are presented for negative relative to positive/close margins, and threshold distances relative to > 0 or 1 mm.

#### Bayesian models (network meta-analysis).

Network meta-analysis using Mixed Treatment Comparisons used data from single or multiple thresholds within studies (when presented) to compare directly (within study) and indirectly (between studies) the probability of LR between margins categories (positive; > 0 or 1 mm; 2 mm; 3 mm; 10 mm). ORs compare negative versus positive margins, and distance categories relative to positive margins.

#### Assessment of covariates.

All models were adjusted for study-level follow up time. Other covariates were assessed for their effect on model estimates (age; median year of recruitment; proportion of patients who received endocrine therapy; proportion with high-grade DCIS; proportion of patients receiving whole breast radiation).

## References

[1] Correa C, McGale P, Taylor C (2010). Overview of the randomized trials of radiotherapy in ductal carcinoma in situ of the breast. J Natl Cancer Inst Monogr.

[2] Narod SA, Iqbal J, Giannakeas V (2015). Breast cancer mortality after a diagnosis of ductal carcinoma in situ. JAMA Oncol.

[3] Worni M, Akushevich I, Greenup R (2015). Trends in treatment patterns and outcomes for ductal carcinoma in situ. J Natl Cancer Inst.

[4] McCormick B, Winter K, Hudis C (2015). RTOG 9804: A prospective randomized trial for good-risk ductal carcinoma in situ comparing radiotherapy with observation. J Clin Oncol.

[5] Houghton J, George WD, Cuzick J (2003). Radiotherapy and tamoxifen in women with completely excised ductal carcinoma in situ of the breast in the UK, Australia, and New Zealand: Randomised controlled trial. Lancet.

[6] Julien JP, Bijker N, Fentiman IS (2000). Radiotherapy in breast-conserving treatment for ductal carcinoma in situ: First results of the EORTC randomised phase III trial 10853. Lancet.

[7] Wapnir IL, Dignam JJ, Fisher B (2011). Long-term outcomes of invasive ipsilateral breast tumor recurrences after lumpectomy in NSABP B-17 and B-24 randomized clinical trials for DCIS. J Natl Cancer Inst.

[8] Emdin SO, Granstrand B, Ringberg A (2006). SweDCIS: Radiotherapy after sector resection for ductal carcinoma in situ of the breast. Results of a randomised trial in a population offered mammography screening. Acta Oncol.

[9] Silverstein MJ, Lagios MD, Groshen S (1999). The influence of margin width on local control of ductal carcinoma in situ of the breast. N Engl J Med.

[10] Azu M, Abrahamse P, Katz SJ (2010). What is an adequate margin for breast-conserving surgery? Surgeon attitudes and correlates. Ann Surg Oncol.

[11] Morrow M, Jagsi R, Alderman AK (2009). Surgeon recommendations and receipt of mastectomy for treatment of breast cancer. JAMA.

[12] King TA, Sakr R, Patil S (2011). Clinical management factors contribute to the decision for contralateral prophylactic mastectomy. J Clin Oncol.

[13] Subhedar P, Olcese C, Patil S, et al: Decreasing recurrence rates for ductal carcinoma in situ: Analysis of 2996 women treated with breast-conserving surgery over 30 years. Ann Surg Oncol 22:3273-3281, 2015 [Erratum: Ann Surg Oncol 22:S1618, 2015] 10.1245/s10434-015-4740-8 PMC4694562 26215193

[14] Greenfield S, Steinberg EP, Auerbach A Clinical practice guidelines we can trust. http://www.nationalacademies.org/hmd/Reports/2011/Clinical-Practice-Guidelines-We-Can-Trust.aspx.

[15] Marinovich ML, Azizi L, Macaskill P (in press). The association of surgical margins and local recurrence in women with ductal carcinoma in situ treated with breast-conserving therapy: A meta-analysis. Ann Surg Oncol.

[16] Bland JM, Altman DG (1998). Bayesians and frequentists. BMJ.

[17] Fisher ER, Costantino J, Fisher B (1995). Pathologic findings from the National Surgical Adjuvant Breast Project (NSABP) protocol B-17. Intraductal carcinoma (ductal carcinoma in situ). Cancer.

[18] Donker M, Litière S, Werutsky G (2013). Breast-conserving treatment with or without radiotherapy in ductal carcinoma in situ: 15-year recurrence rates and outcome after a recurrence, from the EORTC 10853 randomized phase III trial. J Clin Oncol.

[19] Faverly DR, Burgers L, Bult P (1994). Three dimensional imaging of mammary ductal carcinoma in situ: Clinical implications. Semin Diagn Pathol.

[20] Guidi AJ, Connolly JL, Harris JR (1997). The relationship between shaved margin and inked margin status in breast excision specimens. Cancer.

[21] Carter D (1986). Margins of “lumpectomy” for breast cancer. Hum Pathol.

[22] Van Zee KJ, Subhedar P, Olcese C (2015). Relationship between margin width and recurrence of ductal carcinoma in situ: Analysis of 2996 women treated with breast-conserving surgery for 30 years. Ann Surg.

[23] Solin LJ, Gray R, Hughes LL (2015). Surgical excision without radiation for ductal carcinoma in situ of the breast: 12-year results from the ECOG-ACRIN E5194 study. J Clin Oncol.

[24] Wong JS, Chen YH, Gadd MA (2014). Eight-year update of a prospective study of wide excision alone for small low- or intermediate-grade ductal carcinoma in situ (DCIS). Breast Cancer Res Treat.

[25] Cuzick J, Sestak I, Pinder SE (2011). Effect of tamoxifen and radiotherapy in women with locally excised ductal carcinoma in situ: Long-term results from the UK/ANZ DCIS trial. Lancet Oncol.

[26] Bijker N, Meijnen P, Peterse JL (2006). Breast-conserving treatment with or without radiotherapy in ductal carcinoma-in-situ: Ten-year results of European Organisation for Research and Treatment of Cancer randomized phase III trial 10853--a study by the EORTC Breast Cancer Cooperative Group and EORTC Radiotherapy Group. J Clin Oncol.

[27] Pinder SE, Duggan C, Ellis IO (2010). A new pathological system for grading DCIS with improved prediction of local recurrence: Results from the UKCCCR/ANZ DCIS trial. Br J Cancer.

[28] Rakovitch E, Nofech-Mozes S, Hanna W (2015). A population-based validation study of the DCIS Score predicting recurrence risk in individuals treated by breast-conserving surgery alone. Breast Cancer Res Treat.

[29] Solin LJ, Gray R, Baehner FL (2013). A multigene expression assay to predict local recurrence risk for ductal carcinoma in situ of the breast. J Natl Cancer Inst.

[30] Bartelink H, Maingon P, Poortmans P (2015). Whole-breast irradiation with or without a boost for patients treated with breast-conserving surgery for early breast cancer: 20-year follow-up of a randomised phase 3 trial. Lancet Oncol.

[31] Smith BD, Bentzen SM, Correa CR (2011). Fractionation for whole breast irradiation: an American Society for Radiation Oncology (ASTRO) evidence-based guideline. Int J Radiat Oncol Biol Phys.

[32] Smith BD, Arthur DW, Buchholz TA (2009). Accelerated partial breast irradiation consensus statement from the American Society for Radiation Oncology (ASTRO). Int J Radiat Oncol Biol Phys.

[33] Moran MS, Schnitt SJ, Giuliano AE (2014). Society of Surgical Oncology-American Society for Radiation Oncology consensus guideline on margins for breast-conserving surgery with whole-breast irradiation in stages I and II invasive breast cancer. J Clin Oncol.

[34] Edge SB, Byrd DR, Compton C, et al (eds): American Joint Committee on Cancer (AJCC) Cancer Staging Manual (ed 7). New York, NY, Springer, 2011

[35] Li Y, Zhang S, Wei X (2015). The clinical features and management of women with ductal carcinoma in situ with microinvasion: A retrospective cohort study. Int J Surg.

[36] Parikh RR, Haffty BG, Lannin D (2012). Ductal carcinoma in situ with microinvasion: prognostic implications, long-term outcomes, and role of axillary evaluation. Int J Radiat Oncol Biol Phys.

